# Lophine (2,4,5-triphenyl-1*H*-imidazole)

**DOI:** 10.1107/S1600536809006552

**Published:** 2009-03-06

**Authors:** Diana Yanover, Menahem Kaftory

**Affiliations:** a Schulich Faculty of Chemistry, Technion – Israel Institute of Technology, Haifa 32000, Israel

## Abstract

The title compound, C_21_H_16_N_2_, has been known since 1877. Although the crystal structure of 36 derivatives of lophine are known, the structure of parent compound has remained unknown until now. The three phenyl rings bonded to the imidazole core are not coplanar with the latter, with dihedral angles of 21.4 (3), 24.7 (3), and 39.0 (3)°, respectively, between the phenyl ring planes in the 2-, 4- and 5-positions of the imidazole ring. The mol­ecules are packed in layers running perpendicular to the *b* axis. Although there are acceptor and donor atoms for hydrogen bonds, no such inter­actions are detected in the crystal in contrast to other lophine derivatives.

## Related literature

For background on lophine and its derivatives, see: Fridman *et al.* (2008 ▶); Fridman, Kaftory & Speiser (2007 ▶); Fridman, Kaftory, Eichen & Speiser (2007 ▶); Kamidate *et al.* (1989 ▶); Liu *et al.* (2005 ▶); Nakashima (2003 ▶); Nakashima *et al.* (1995 ▶); Radziszewski (1877 ▶); Seethalakshmi *et al.* (2006 ▶); Thiruvalluvar *et al.* (2007 ▶); Thuer *et al.* (2004 ▶). For information about the Cambridge Database, see: Allen (2002 ▶). For related literature, see: Inouye & Sakaino (2000 ▶); Kaftory *et al.* (1998 ▶); Santos *et al.* (2001 ▶).
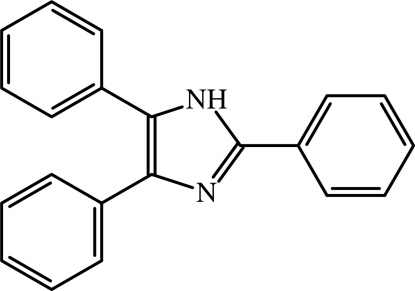


## Experimental

### 

#### Crystal data

C_21_H_16_N_2_
*M* *_r_* = 296.36
Orthorhombic, 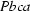

*a* = 20.218 (4) Å
*b* = 7.538 (2) Å
*c* = 20.699 (4) Å
*V* = 3154.6 (12) Å^3^
*Z* = 8
Mo *K*α radiation
μ = 0.07 mm^−1^
*T* = 293 K
0.50 × 0.10 × 0.05 mm

#### Data collection

Nonius KappaCCD diffractometer
Absorption correction: none
22192 measured reflections
2747 independent reflections
1036 reflections with *I* > 2σ(*I*)
*R*_int_ = 0.143

#### Refinement

*R*[*F* ^2^ > 2σ(*F* ^2^)] = 0.088
*wR*(*F* ^2^) = 0.245
*S* = 1.09
2747 reflections
209 parameters
H-atom parameters constrained
Δρ_max_ = 0.25 e Å^−3^
Δρ_min_ = −0.30 e Å^−3^

### 

Data collection: *COLLECT* (Nonius, 2006); cell refinement: *DENZO* *HKL-2000* (Otwinowski & Minor, 1997 ▶); data reduction: *DENZO* *HKL-2000*; program(s) used to solve structure: *SHELXS97* (Sheldrick, 2008 ▶); program(s) used to refine structure: *SHELXL97* (Sheldrick, 2008 ▶); molecular graphics: *ORTEP-3 for Windows* (Farrugia, 1999 ▶); software used to prepare material for publication: *SHELXL97*.

## Supplementary Material

Crystal structure: contains datablocks global, I. DOI: 10.1107/S1600536809006552/hb2915sup1.cif

Structure factors: contains datablocks I. DOI: 10.1107/S1600536809006552/hb2915Isup2.hkl

Additional supplementary materials:  crystallographic information; 3D view; checkCIF report

## Figures and Tables

**Fig. 1.**
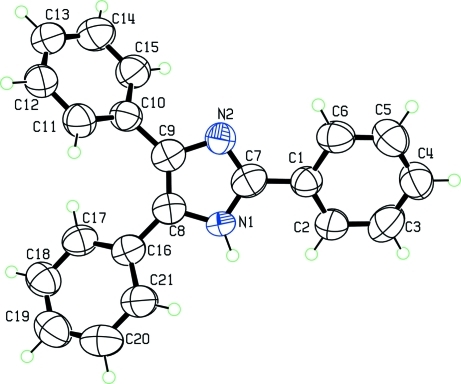
Molecular structure of (I). Displacement ellipsoids are drawn at the 50% probability level.

**Fig. 2.**
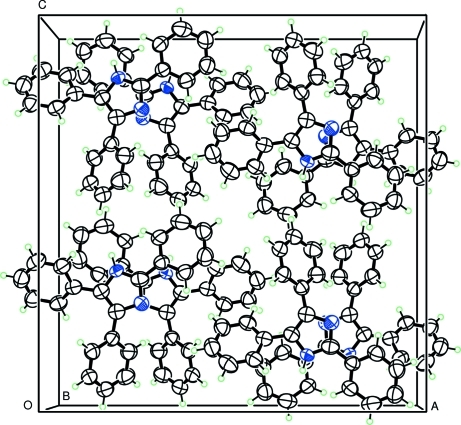
Unit cell packing for (I).

## supplementary crystallographic information

### Comment

Recently, heterocyclic imidazole derivatives have attracted considerable attention because of their unique optical properties (Santos *et al.*, 1996). These compounds play a very important role in chemistry as multipurpose analytical tools (Nakashima *et al.*, 1995 & Nakashima, 2003 & Kamidate *et al.*, 1989). Lophine (2,4,5-triphenyl-1*H*-imidazole) (I) is an attractive fluorescence and chemiluminescence compound. The chemiluminescence properties of this synthetic organic compound was reported for the first time by Radziszewski (1877). He showed that lophine emits a yellow light when it reacts with oxygen in the presence of a strong base. Since then many lophine derivatives have been synthesized and studied with regards to their optical properties. Lophine was chosen as the molecule of the week (December 15, 2008) by the American Chemical Society with the following summary "Lophine (2,4,5-triphenyl-1*H*-imidazole) exhibits lemon yellow chemiluminescence in solution and is one of the few long-lasting chemiluminescence molecules. It forms dimmers that have piezochromic and photochromic properties. It has been proposed as an analytical reagent for trace metal ion detection." The crystal structure of 36 different lophine derivatives have been deposited at the Cambridge Crystallographic Data Center (Allen, 2002). It is interesting to note, however, that the crystal structure of the parent lophine compound was never published. We undertook a search for new lophine derivatives that will show thermo- and photochromic properties (Fridman *et al.*, 2007, Fridman *et al.*, 2007, Fridman *et al.*, 2008).

During our investigation we have succeeded to identify crystals of neat lophine. Here we describe its crystal structure. The molecular structure of lophine depicted in Figure 1, may be described by the rotation of three phenyl rings about their bonds to the imidazole ring. The rotation angles of the phenyl rings plane are 21.4, 24.7, and 39.0° at the 2, 4 and 5 positions of the imidazole ring respectively. Although the imidazole ring bearing H-donor and H-acceptor N atoms, they do not participate in hydrogen bonding as found in five other lophine derivatives (Seethalakshmi *et al.*, 2006, Ynouye & Sakaino, 1986, Thuer *et al.*, 2004, Liu *et al.*, 2005, Thiruvalluvar *et al.*, 2007) where the lophine derivative molecules packed in chains made up of hydrogen bonded molecules of N—H···N type. All other lophine derivatives form hydrogen bonds through the N—H or the N atoms either with molecules of the same kind or with solvent molecules. The absent of strong intermolecular forces might be the reason for the lack of published crystal structure of neat lophine because it is difficult to crystallize it.

### Experimental

Lophine synthesis: benzil (1 mmol), suitable benzaldehyde (1 mmol), and ammonium acetate (1.2 g) were dissolved in boiling glacial acetic acid (16 ml) and refluxed for 5–24 h. The reaction progress was monitored by TLC. After the reaction completion, the reaction mixture was poured into ice-water, washed with NaHCO_3_ and then washed several times with EtOAc. The combined extracts were dried over MgSO_4_. The purification was done by flash column chromatography. The compound was obtained in 46% yield. Colourless plates of (I) were recrystallised from DMSO.

### Refinement

The quality of the crystals was poor which is also reflected in the crystal structure refinement. H atoms were clearly found in the difference Fourier maps. All H atoms were refined at idealized positions riding on the C and N atoms with C—H = 0.96 Å, and N—H = 0.86 Å, and *U*_iso_(H) = 1.2*U*_eq_(C or N).

### Crystal data

**Table:**

C_21_H_16_N_2_	*F*(000) = 1248
*M**_r_* = 296.36	*D*_x_ = 1.248 Mg m^−^^3^
Orthorhombic, *P**b**c**a*	Mo *K*α radiation, λ = 0.71073 Å
Hall symbol: -P 2ac 2ab	Cell parameters from 22192 reflections
*a* = 20.218 (4) Å	θ = 2.0–25.3°
*b* = 7.538 (2) Å	µ = 0.07 mm^−^^1^
*c* = 20.699 (4) Å	*T* = 293 K
*V* = 3154.6 (12) Å^3^	Plate, colorless
*Z* = 8	0.50 × 0.10 × 0.05 mm

### Data collection

**Table:**

Nonius KappaCCD diffractometer	1036 reflections with *I* > 2σ(*I*)
Radiation source: fine-focus sealed tube	*R*_int_ = 0.143
graphite	θ_max_ = 25.3°, θ_min_ = 2.0°
φ and ω scans	*h* = −23→22
22192 measured reflections	*k* = −8→8
2747 independent reflections	*l* = −24→23

### Refinement

**Table:**

Refinement on *F*^2^	Secondary atom site location: difference Fourier map
Least-squares matrix: full	Hydrogen site location: inferred from neighbouring sites
*R*[*F*^2^ > 2σ(*F*^2^)] = 0.088	H-atom parameters constrained
*wR*(*F*^2^) = 0.245	*w* = 1/[σ^2^(*F*_o_^2^) + (0.0863*P*)^2^] where *P* = (*F*_o_^2^ + 2*F*_c_^2^)/3
*S* = 1.09	(Δ/σ)_max_ < 0.001
2747 reflections	Δρ_max_ = 0.25 e Å^−^^3^
209 parameters	Δρ_min_ = −0.30 e Å^−^^3^
0 restraints	Extinction correction: *SHELXL97* (Sheldrick, 2008), Fc^*^=kFc[1+0.001xFc^2^λ^3^/sin(2θ)]^-1/4^
Primary atom site location: structure-invariant direct methods	Extinction coefficient: 0.0089 (18)

### Special details

**Table:**

Geometry. All e.s.d.'s (except the e.s.d. in the dihedral angle between two l.s. planes) are estimated using the full covariance matrix. The cell e.s.d.'s are taken into account individually in the estimation of e.s.d.'s in distances, angles and torsion angles; correlations between e.s.d.'s in cell parameters are only used when they are defined by crystal symmetry. An approximate (isotropic) treatment of cell e.s.d.'s is used for estimating e.s.d.'s involving l.s. planes.
Refinement. Refinement of *F*^2^ against ALL reflections. The weighted *R*-factor *wR* and goodness of fit *S* are based on *F*^2^, conventional *R*-factors *R* are based on *F*, with *F* set to zero for negative *F*^2^. The threshold expression of *F*^2^ > σ(*F*^2^) is used only for calculating *R*-factors(gt) *etc*. and is not relevant to the choice of reflections for refinement. *R*-factors based on *F*^2^ are statistically about twice as large as those based on *F*, and *R*- factors based on ALL data will be even larger.

### Fractional atomic coordinates and isotropic or equivalent isotropic displacement parameters (Å^2^)

**Table:**

	*x*	*y*	*z*	*U*_iso_*/*U*_eq_	
N1	0.6928 (2)	0.1996 (5)	0.14220 (18)	0.0556 (11)	
H1	0.6809	0.1806	0.1029	0.067*	
N2	0.7563 (2)	0.2458 (5)	0.2252 (2)	0.0668 (12)	
C1	0.8110 (3)	0.1974 (7)	0.1209 (3)	0.0660 (14)	
C2	0.8052 (3)	0.2208 (9)	0.0564 (3)	0.0928 (19)	
H2	0.7620	0.2437	0.0390	0.111*	
C3	0.8616 (4)	0.2073 (9)	0.0170 (3)	0.108 (2)	
H3	0.8570	0.2267	−0.0286	0.129*	
C4	0.9225 (3)	0.1634 (8)	0.0422 (3)	0.0890 (19)	
H4	0.9611	0.1517	0.0155	0.107*	
C5	0.9267 (3)	0.1408 (8)	0.1067 (4)	0.0902 (19)	
H5	0.9694	0.1134	0.1244	0.108*	
C6	0.8727 (3)	0.1565 (7)	0.1470 (3)	0.0822 (18)	
H6	0.8778	0.1408	0.1927	0.099*	
C7	0.7536 (3)	0.2141 (7)	0.1634 (3)	0.0767 (16)	
C8	0.6519 (3)	0.2205 (6)	0.1946 (3)	0.0672 (14)	
C9	0.6911 (3)	0.2496 (6)	0.2465 (3)	0.0690 (14)	
C10	0.6777 (3)	0.2762 (7)	0.3158 (2)	0.0689 (15)	
C11	0.6295 (3)	0.1845 (7)	0.3489 (3)	0.0771 (16)	
H11	0.6028	0.1011	0.3255	0.093*	
C12	0.6206 (3)	0.2137 (9)	0.4140 (3)	0.0876 (18)	
H12	0.5870	0.1509	0.4375	0.105*	
C13	0.6578 (4)	0.3288 (10)	0.4475 (3)	0.098 (2)	
H13	0.6503	0.3469	0.4928	0.118*	
C14	0.7082 (4)	0.4240 (10)	0.4148 (3)	0.098 (2)	
H14	0.7355	0.5090	0.4367	0.118*	
C15	0.7179 (3)	0.3959 (8)	0.3504 (3)	0.0841 (18)	
H15	0.7531	0.4548	0.3279	0.101*	
C16	0.5797 (3)	0.2127 (7)	0.1838 (2)	0.0673 (15)	
C17	0.5352 (3)	0.3003 (7)	0.2245 (3)	0.0739 (16)	
H17	0.5523	0.3636	0.2611	0.089*	
C18	0.4681 (3)	0.2945 (8)	0.2130 (3)	0.0827 (17)	
H18	0.4383	0.3568	0.2411	0.099*	
C19	0.4436 (3)	0.2035 (9)	0.1613 (3)	0.0944 (19)	
H19	0.3968	0.1983	0.1536	0.113*	
C20	0.4872 (4)	0.1191 (8)	0.1199 (3)	0.095 (2)	
H20	0.4697	0.0557	0.0835	0.114*	
C21	0.5545 (3)	0.1206 (7)	0.1311 (3)	0.0805 (17)	
H21	0.5846	0.0615	0.1024	0.097*	

### Atomic displacement parameters (Å^2^)

**Table:**

	*U*^11^	*U*^22^	*U*^33^	*U*^12^	*U*^13^	*U*^23^
N1	0.056 (3)	0.067 (2)	0.044 (2)	0.000 (2)	−0.008 (2)	−0.0054 (19)
N2	0.065 (3)	0.072 (3)	0.064 (3)	−0.005 (2)	0.003 (3)	0.005 (2)
C1	0.069 (4)	0.066 (3)	0.062 (4)	0.004 (3)	−0.003 (3)	0.001 (3)
C2	0.077 (5)	0.133 (5)	0.068 (4)	0.024 (4)	−0.010 (4)	−0.006 (4)
C3	0.120 (6)	0.138 (6)	0.065 (4)	0.011 (5)	0.008 (4)	−0.004 (4)
C4	0.076 (5)	0.097 (4)	0.094 (5)	0.009 (3)	0.005 (4)	0.007 (4)
C5	0.074 (5)	0.094 (4)	0.102 (5)	0.003 (3)	−0.003 (4)	0.018 (4)
C6	0.085 (5)	0.087 (4)	0.074 (4)	−0.016 (3)	−0.011 (4)	0.012 (3)
C7	0.094 (5)	0.068 (3)	0.068 (4)	−0.003 (3)	−0.002 (4)	0.008 (3)
C8	0.074 (4)	0.060 (3)	0.068 (4)	0.002 (3)	−0.005 (3)	0.003 (3)
C9	0.082 (4)	0.056 (3)	0.070 (4)	−0.004 (3)	−0.002 (3)	0.000 (3)
C10	0.066 (4)	0.072 (3)	0.068 (4)	−0.005 (3)	−0.006 (3)	0.000 (3)
C11	0.070 (4)	0.083 (4)	0.077 (4)	−0.011 (3)	0.000 (3)	0.008 (3)
C12	0.069 (4)	0.122 (5)	0.071 (4)	0.003 (4)	−0.003 (4)	0.021 (4)
C13	0.081 (5)	0.146 (6)	0.067 (4)	0.015 (4)	−0.006 (4)	−0.010 (4)
C14	0.102 (6)	0.108 (5)	0.084 (5)	−0.004 (4)	−0.012 (4)	−0.021 (4)
C15	0.086 (4)	0.090 (4)	0.077 (4)	−0.018 (3)	−0.001 (3)	0.000 (3)
C16	0.081 (4)	0.059 (3)	0.062 (3)	−0.008 (3)	−0.004 (3)	0.002 (3)
C17	0.086 (5)	0.067 (3)	0.068 (4)	0.006 (3)	−0.010 (3)	0.001 (3)
C18	0.075 (5)	0.083 (4)	0.090 (5)	0.003 (3)	−0.006 (4)	0.012 (4)
C19	0.075 (5)	0.104 (5)	0.105 (5)	−0.005 (4)	−0.012 (4)	0.020 (4)
C20	0.106 (6)	0.090 (4)	0.090 (5)	−0.016 (4)	−0.027 (5)	−0.004 (4)
C21	0.086 (5)	0.083 (4)	0.072 (4)	−0.009 (3)	−0.010 (4)	0.000 (3)

### Geometric parameters (Å, °)

**Table:**

N1—C7	1.310 (7)	C10—C15	1.410 (7)
N1—C8	1.374 (6)	C11—C12	1.377 (7)
N1—H1	0.8600	C11—H11	0.9600
N2—C7	1.302 (6)	C12—C13	1.342 (8)
N2—C9	1.391 (6)	C12—H12	0.9600
C1—C2	1.353 (7)	C13—C14	1.419 (9)
C1—C6	1.392 (7)	C13—H13	0.9600
C1—C7	1.462 (7)	C14—C15	1.363 (7)
C2—C3	1.406 (8)	C14—H14	0.9600
C2—H2	0.9602	C15—H15	0.9599
C3—C4	1.378 (8)	C16—C17	1.398 (7)
C3—H3	0.9600	C16—C21	1.390 (7)
C4—C5	1.348 (7)	C17—C18	1.377 (7)
C4—H4	0.9600	C17—H17	0.9602
C5—C6	1.378 (7)	C18—C19	1.364 (8)
C5—H5	0.9600	C18—H18	0.9599
C6—H6	0.9602	C19—C20	1.384 (9)
C8—C9	1.353 (7)	C19—H19	0.9600
C8—C16	1.477 (7)	C20—C21	1.380 (8)
C9—C10	1.474 (7)	C20—H20	0.9601
C10—C11	1.377 (7)	C21—H21	0.9599
			
C7—N1—C8	107.0 (4)	C12—C11—C10	119.9 (6)
C7—N1—H1	126.5	C12—C11—H11	121.6
C8—N1—H1	126.5	C10—C11—H11	118.5
C7—N2—C9	106.0 (5)	C13—C12—C11	122.4 (6)
C2—C1—C6	119.3 (6)	C13—C12—H12	117.0
C2—C1—C7	120.9 (6)	C11—C12—H12	120.6
C6—C1—C7	119.8 (5)	C12—C13—C14	118.9 (6)
C1—C2—C3	119.5 (6)	C12—C13—H13	120.6
C1—C2—H2	118.3	C14—C13—H13	120.6
C3—C2—H2	122.2	C15—C14—C13	119.4 (6)
C4—C3—C2	121.5 (6)	C15—C14—H14	118.9
C4—C3—H3	119.7	C13—C14—H14	121.6
C2—C3—H3	118.8	C14—C15—C10	120.9 (6)
C5—C4—C3	117.5 (6)	C14—C15—H15	120.6
C5—C4—H4	120.5	C10—C15—H15	118.5
C3—C4—H4	122.0	C17—C16—C21	118.2 (5)
C4—C5—C6	122.5 (6)	C17—C16—C8	121.7 (5)
C4—C5—H5	117.4	C21—C16—C8	120.1 (5)
C6—C5—H5	120.0	C18—C17—C16	121.0 (5)
C5—C6—C1	119.6 (6)	C18—C17—H17	120.3
C5—C6—H6	120.0	C16—C17—H17	118.7
C1—C6—H6	120.4	C19—C18—C17	120.5 (6)
N1—C7—N2	112.5 (6)	C19—C18—H18	119.5
N1—C7—C1	122.4 (5)	C17—C18—H18	120.0
N2—C7—C1	125.0 (6)	C18—C19—C20	119.1 (6)
C9—C8—N1	107.0 (5)	C18—C19—H19	120.6
C9—C8—C16	134.8 (6)	C20—C19—H19	120.4
N1—C8—C16	118.1 (5)	C21—C20—C19	121.4 (6)
C8—C9—N2	107.5 (5)	C21—C20—H20	119.9
C8—C9—C10	133.5 (6)	C19—C20—H20	118.7
N2—C9—C10	119.0 (5)	C20—C21—C16	119.8 (6)
C11—C10—C15	118.5 (5)	C20—C21—H21	121.3
C11—C10—C9	123.1 (5)	C16—C21—H21	118.9
C15—C10—C9	118.4 (5)		
			
C6—C1—C2—C3	−1.4 (9)	C8—C9—C10—C11	38.5 (8)
C7—C1—C2—C3	178.7 (6)	N2—C9—C10—C11	−139.1 (5)
C1—C2—C3—C4	2.7 (10)	C8—C9—C10—C15	−144.2 (6)
C2—C3—C4—C5	−2.6 (10)	N2—C9—C10—C15	38.2 (7)
C3—C4—C5—C6	1.3 (9)	C15—C10—C11—C12	1.4 (9)
C4—C5—C6—C1	−0.1 (9)	C9—C10—C11—C12	178.7 (5)
C2—C1—C6—C5	0.1 (8)	C10—C11—C12—C13	−0.5 (10)
C7—C1—C6—C5	180.0 (5)	C11—C12—C13—C14	0.0 (10)
C8—N1—C7—N2	−1.1 (6)	C12—C13—C14—C15	−0.3 (10)
C8—N1—C7—C1	179.4 (5)	C13—C14—C15—C10	1.2 (10)
C9—N2—C7—N1	1.0 (6)	C11—C10—C15—C14	−1.8 (9)
C9—N2—C7—C1	−179.5 (5)	C9—C10—C15—C14	−179.2 (6)
C2—C1—C7—N1	20.7 (8)	C9—C8—C16—C17	24.5 (9)
C6—C1—C7—N1	−159.2 (5)	N1—C8—C16—C17	−153.3 (5)
C2—C1—C7—N2	−158.7 (6)	C9—C8—C16—C21	−157.2 (5)
C6—C1—C7—N2	21.4 (8)	N1—C8—C16—C21	25.0 (7)
C7—N1—C8—C9	0.7 (5)	C21—C16—C17—C18	0.5 (8)
C7—N1—C8—C16	179.1 (4)	C8—C16—C17—C18	178.8 (4)
N1—C8—C9—N2	−0.1 (5)	C16—C17—C18—C19	−0.3 (8)
C16—C8—C9—N2	−178.1 (5)	C17—C18—C19—C20	−0.9 (9)
N1—C8—C9—C10	−177.9 (5)	C18—C19—C20—C21	2.0 (10)
C16—C8—C9—C10	4.1 (10)	C19—C20—C21—C16	−1.9 (9)
C7—N2—C9—C8	−0.5 (5)	C17—C16—C21—C20	0.6 (8)
C7—N2—C9—C10	177.6 (4)	C8—C16—C21—C20	−177.7 (5)

## References

[bb1] Allen, F. H. (2002). *Acta Cryst.* B**58**, 380–388. 10.1107/s0108768102003890 12037359

[bb2] Farrugia, L. J. (1999). *J. Appl. Cryst.* **32**, 837–838.

[bb3] Fridman, N., Kaftory, M., Eichen, Y. & Speiser, S. (2007). J. Photochem. Photobiol. A, 188, 25-33.

[bb4] Fridman, N., Kaftory, M., Eichen, Y. & Speiser, S. (2008). *J. Mol. Struct.* **917**, 101-109.

[bb5] Fridman, N., Kaftory, M. & Speiser, S. (2007). *Sens. Actuators*, **B126**, 107-115.

[bb6] Inouye, Y. & Sakaino, Y. (2000). *Acta Cryst.* C**56**, 884–887. 10.1107/s0108270100005205 10935117

[bb7] Kaftory, M., Taycher, H. & Botoshansky, M. (1998). *J. Chem. Soc. Perkin Trans. 2*, pp. 407-412.

[bb8] Kamidate, T., Yamaguchi, K., Segawa, T. & Watanabe, H. (1989). *Anal. Sci* **5**, 429-33.

[bb9] Liu, X.-F., Zhong, Z.-P. & Xu, Z.-L. (2005). *Acta Cryst.* E**61**, o1976–o1977.

[bb10] Nakashima, K. (2003). *Biomed. Chromatogr* **17**, 83-95. 10.1002/bmc.226 12717796

[bb11] Nakashima, K., Yamasaki, H., Kuroda, N. & Akiyama, S. (1995). *Anal. Chim. Acta*, **303**, 103-107.

[bb12] Nonius (2000). *COLLECT* Nonius BV, Delft, The Netherlands.

[bb13] Otwinowski, Z. & Minor, W. (1997). *Methods in Enzymology*, Vol. 276, Macromolecular Crystallography, Part A, edited by C. W. Carter Jr & R. M. Sweet, pp. 307-326. New York: Academic Press.

[bb14] Radziszewski, B. (1877). *Chem. Ber* **10**, 70-75.

[bb15] Santos, J., Mintz, E. A., Zehnder, O., Bosshard, C., Bu, X. R. & Gunter, P. (2001). *Tetrahedron Lett* **42**, 805-808.

[bb16] Seethalakshmi, T., Puratchikody, A., Lynch, D. E., Kaliannan, P. & Thamotharan, S. (2006). *Acta Cryst.* E**62**, o2803–o2804.

[bb17] Sheldrick, G. M. (2008). *Acta Cryst.* A**64**, 112–122. 10.1107/S0108767307043930 18156677

[bb18] Thiruvalluvar, A., Balamurugan, S., Puratchikody, A. & Nallu, M. (2007). *Acta Cryst.* E**63**, o1650–o1652.

[bb19] Thuer, W., Gompper, R. & Polborn, K. (2004). Private communication (deposition CCDC 259545). CCDC, Cambridge, England.

